# A Green Approach Towards Desalination: Sustainable Poly(lactic acid) Membranes for Pervaporation Desalination

**DOI:** 10.3390/membranes16060206

**Published:** 2026-06-10

**Authors:** Urooj Ahmad, Bart Van der Bruggen, Xing Yang

**Affiliations:** Department of Chemical Engineering, KU Leuven, Celestijnenlaan 200F, B-3001 Leuven, Belgium; urooj.ahmad@kuleuven.be (U.A.); xing.yang@kuleuven.be (X.Y.)

**Keywords:** desalination, membranes, pervaporation, green chemistry, poly(lactic acid)

## Abstract

To address the global water crisis, desalination technologies contribute about 1% of the global freshwater supply. Membrane-based desalination technologies offer high performance, operational ease, cost-effectiveness and high scalability compared to conventional thermal desalination modes. Among all membrane-based technologies, reverse osmosis is prevailing globally. However, the high energy demand of the reverse osmosis process and fouling in case of hypersaline feed streams motivate the exploration of alternative technologies, i.e., pervaporation. Pervaporation desalination involves dense hydrophilic polymer membranes to deal with high salt streams at low cost, along with less fouling than a few other membrane processes, i.e., reverse osmosis and membrane distillation. Mass transport through pervaporation desalination membranes is well-explained by solution-diffusion theory involving a tri-stage transfer, i.e., sorption, diffusion and evaporation. Since the last few decades, a green approach in all domains has offered chemical products and processes with the least hazards and minimal waste production. Application of biodegradable materials like poly(lactic acid) in combination with suitable green solvents, e.g., ethyl lactate, methyl lactate, cyrene, dimethyl isosorbide and gamma valerolactone for pervaporation desalination would be a good roadmap to meet the sustainability criterion. Some intrinsic features of poly(lactic acid) that make it a ‘material of choice’ for pervaporation desalination include hydrophilicity imparted by the presence of polar ester groups, high salt rejection, biodegradability with simple mineralization products, i.e., H_2_O and CO_2_, sustainable production, low toxicity, low carbon footprint, ease of processing and versatility. Poly(lactic acid) undergoes four interrelated degradation mechanisms: hydrolytic degradation, biodegradation, thermal degradation and photodegradation. The concern for poly(lactic acid) based pervaporation desalination is increased hydrolytic cleavage of poly(lactic acid) at high temperatures, which requires some modifications, e.g., nanoenhancement, additions of crosslinkers, surface modifications, addition of other polymers to prepare blends and post-treatments. These modifying strategies result in an increased stability and better performance of poly(lactic acid) films. However, optimization of various parameters relevant to such modifications leaves room for further research. This review offers a critical analysis of the need for biodegradable polymers with special focus on poly(lactic acid) rather than their fossil fuel-based alternatives, the environmental and health effects of all these polymers, cost estimation and possible performance-efficient, green and eco-friendly solutions.

## 1. Introduction

An evident switch from a linear to a circular economy needs a practical implementation in the technical domain instead of being limited to a theoretical abstraction [1]. There are multiple detrimental outcomes of excessively applied hazardous chemicals (i.e., notable climatic variations in the recent decades, depletion of resources, energy crisis and worldwide pandemics) that depict the loopholes of present-day chemistry and demand modifications in production modes [2,3]. In this regard, life-favourable production of chemical goods plays a significant role, which reduces the health risks to living beings and also protects the ecosystem in a sustainable civilization [4]. Since the last few decades, the attempts in the domain of Green Chemistry have promoted the development of chemical agents and procedures with minimal hazards and waste production [5]. The Organization for Economic Co-operation and Development (OECD) encouraged the concept of sustainable chemistry, which involves an innovation in production modes and goods, managing policies, better performance and added value, minimal health risks and ecosystem protection. Nowadays, the extended criterion of sustainability includes conservation of resources, resource recovery and recycling, and full life-cycle assessment of the goods, which encourages the green and healthy planet goals [6].

The United Nations’ 2030 Agenda for Sustainable Development has included the ‘Water Goal’, highlighting the significance of a sustainable supply of water for drinking and sanitation [7]. Based on a recent estimation by the World Health Organization (WHO), around 785 million people are facing a shortage of potable water [8], and those living in water-scarce regions are estimated to be more than 2000 million [9]. The crucial factors involved in declining water reserves are climatic variations [8,10], urbanization, population explosion, industrialization [9], aquatic pollution [10,11] and groundwater exploitation [11]. Henceforth, it is crucial to produce freshwater in order to cope with the requirements for average living standards. Desalination, as a remarkable mode to deal with water scarcity, provides around 1% of global freshwater and meets the water needs of 4% of the global population (300 million people) [12]. To address the problem of freshwater and increasing demands for drinking and irrigation purposes, desalination technologies play a crucial role in harnessing seawater as a sustainable resource [13,14]. Other than natural saline water streams, including brackish water and seawater, anthropogenic saline water streams also exist, such as industrial wastewater, agricultural runoff based on salty groundwater, and desalination concentrates [15].

Amongst many desalination technologies, membrane-based technologies are emerging and have demonstrated certain advantages, i.e., higher selectivity with comparatively mild operational parameters, reduced energy costs and operational simplicity leading to the ease of scaling-up. Wider adoption of membrane technology is evident as 70% of the global desalination capacity is relying on membrane technology [16,17]. Besides process parameters, the choice of material for the synthesis of the membrane plays a crucial role in its performance. While selecting a specific material, the inherent physical and chemical features of the material to be considered are its permeation and selection towards the targeted species, mechanical strength, thermal and chemical resistance, and economic and operational feasibility in the given set of conditions [18,19].

On the basis of the chemical composition of the chosen material, membranes can be classified as organic (glassy or rubbery polymers) [20], inorganic (ceramic, hydroxyapatite, etc.) and hybrid membranes [20,21]. Among the membrane-based desalination technologies, reverse osmosis is the most popular one. In reverse osmosis, by the involvement of external pressure, water is pushed from higher to lower solute concentrations through a semi-permeable membrane of pore size in the range of 0.1–1 nm to yield de-mineralized water as permeate [17] and concentrated brine as retentate [22].

The secret of the extensive worldwide trend of reverse osmosis lies in the advantages it offers, such as wide spectrum applicability irrespective of the nature of the water source, high selectivity for the specific contaminants, excellent salt rejection, considerably long lifespan of the membrane, and minimal usage of chemicals [23,24,25]. However, the too high energy demand (specifically at higher salt concentrations of feed streams) is the decisive factor for switching from reverse osmosis to more advanced and economically feasible technologies. Between 30 and 50% of the total operational cost for reverse osmosis serves only for external pressure or pumping to oppose the natural osmotic pressure [17]. Moreover, the fouling of the membrane plays a significant role in lowering the permeate flux, which in turn raises the pumping demands and overall operational cost [26]. In the past few decades, membrane-based desalination technologies utilizing thermal energy, namely, membrane distillation with hydrophobic membranes and pervaporation with hydrophilic membranes, have been in the spotlight [27,28]. This is because of their unique features of considerably high separation performance (above 99.9%) in one-step desalination operation and low energy expense in comparison with other techniques, specifically reverse osmosis [29,30]. In case of highly saline feed streams [31,32], pervaporation and membrane distillation act as alternatives to high-pressure reverse osmosis, as both are independent of osmotic pressure. The pervaporation process, due to its hydrophilic membrane, yields high-purity permeate water [33] and is less prone to fouling [34,35].

In pervaporation, pure water is obtained upon condensation, followed by a phase change without compromising the rejection efficiency of non-volatile species, i.e., salts [15]. The difference in vapour pressure between the feed and permeate streams or chemical potential gradient acts as a driving force in pervaporation [36]. Application of low-grade and renewable energy means (industrial waste heat, geothermal and solar energy) could also be a key for the considerable reduction in energy expenses of pervaporation desalination, while the feed solution is heated in the range of 40–75 °C [37]. The transport through pervaporation membranes occurs through a solution-diffusion mechanism where molecules in the heated feed solution penetrate and diffuse through the membrane on the basis of their chemical affinity, and then the vaporized permeate is received [32,38] and condensed by a cold trap [38,39,40]. On commercial scale, the water permeation flux is considered the decisive factor that remains lower in membrane distillation (2–30 kg/m^2^h with energy input of 0.6–1.75 kWh/m^3^) and pervaporation (0.05–15 kg/m^2^h with energy input of 2 kWh/m^3^) than in reverse osmosis (30–60 kg/m^2^h with energy input of 2–4 kWh/m^3^) which makes the use of economical energy means more significant for phase change in membrane distillation and pervaporation compared to reverse osmosis [39]. Apart from the prime non-wetting advantage of pervaporation desalination [34,35], several other benefits over membrane distillation desalination are reported, for instance, (a) compared to membrane distillation, pervaporation is less prone to temperature polarization due to uncoupled heat optimization and mass transfer, which offers higher salt rejection [41] and increases the performance predictability for hypersaline feed streams at large scale [42,43] (b) for prolonged high temperature applications, dense hydrophilic pervaporation membranes are thermo-mechanically robust and are easily fabricated at low thickness, while less stable hydrophobic microporous membranes for membrane distillation need precise engineering that hinders large-scale applicability [28] (c) the easier design and integration of pervaporation module requiring only standard condensers coupled to simple vacuum driven membrane units increases the chances of scale-up, unlike complex membrane distillation units demanding careful control of thermal gradients, condensation interfaces and heat recovery [28,42].

The materials employed for the synthesis of pervaporation membranes have been modified as per the requirements of the process in order to enhance the performance parameters. In general, the materials with high hydrophilicity are preferable for pervaporation desalination [28]. The availability of materials for pervaporation desalination is almost in all domains of choice including: (a) inorganics (zeolites [44,45,46], silicates [47] etc.), (b) polymers (cellulose and its derivatives [11,48,49], polyether amide [50], poly(vinyl alcohol) [31,51,52], sulfonated polyethylene [53] and polyether ester [54] etc.), and (c) hybrid materials poly(vinyl alcohol) /maleic acid/silica [55], chitosan/graphene oxide [56] etc.). The current trends in research are more oriented towards sustainable waste management, specifically on synthetic polymers, due to their consideration in the United Nations 2030 Agenda for Sustainable Development [57]. Therefore, the application of biodegradable polymers [58,59,60,61] and non-hazardous, green solvents (dimethyl isosorbide, cyrene, gamma valerolactone, ethyl lactate, tamisolve, methyl lactate) [61,62,63,64] rather than the conventional organic solvents (chloroform, dimethylformamide, tetrahydrofuran, toluene, etc.) [65] has become the need of the hour in membrane technology. In this regard, poly(lactic acid) is in hype these days based on its attractive features for pervaporation desalination, including versatility, compostability [66], processability, biodegradability, high mechanical strength, very low toxicity [66,67], bioabsorbability, biocompatibility, high thermal strength, energy efficiency, low carbon footprint and good modulation [67].

This review provides a comparative analysis of various fossil fuel-based and biodegradable polymeric pervaporation membranes based on some crucial factors, i.e., cost, availability and toxic effects on organisms and the ecosystem. Amongst all available choices, the significance of poly(lactic acid) as a promising pervaporation desalination membrane material is highlighted, involving (1) a sustainable membrane synthesis procedure, (2) keys to maintain a critical balance of application-required stability and eco-friendly biodegradability, (3) the role of microplastics and remedies to cope with them, and (4) a cost-comparison with other alternatives. Overall, the current critical analysis contributes a roadmap towards sustainable green pervaporation membrane materials and strategies to cope with the relevant challenges, i.e., cost, eco-friendly synthesis, delicate equilibrium between durability and biodegradability, and toxicity to organisms and the ecosystem.

## 2. Materials Selection for Pervaporation Desalination Membranes

**Figure 1:**
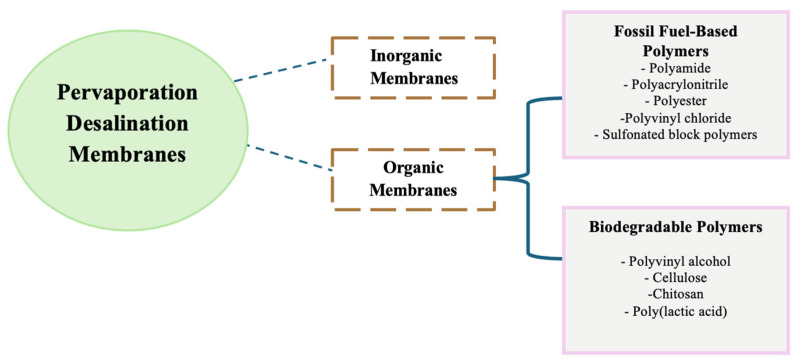
General Classification of Pervaporation Desalination Membranes Based on Choice of Material.

All sorts of materials employed for pervaporation desalination are classified into three major groups, i.e., inorganic, organic and mixed matrix. Organic pervaporation membranes could be composed of fossil fuel-based polymers or biodegradable polymers (Figure 1). On the basis of the choice of material and applicability for specified separations, pervaporation technology is divided into two sub-groups: hydrophobic pervaporation and hydrophilic pervaporation. In hydrophobic pervaporation, selective separation of organic compounds from aqueous mixtures is performed by the application of a hydrophobic membrane [68]. Contrarily, in the case of hydrophilic pervaporation, a hydrophilic membrane material aids in the enhancement of penetration and dispersion of water molecules, e.g., pervaporation desalination membranes [69].

## 3. Pervaporation Membranes Made from Fossil Fuel-Based Polymers

Fossil fuel-based polymers employed for the synthesis of pervaporation desalination membranes include polyamide, polyacrylonitrile and sulphonated block polymers, which are non-biodegradable and are responsible for environmental pollution and notable health hazards. The pollution caused by plastics has reached an alarming level, as depicted by a recent study narrating that out of the total annual global plastic production, 20 million tons is released into the natural environment [70]. Photooxidation and mechanical collision of larger plastic entities yield microplastics [71]. The main polymers that are involved in this kind of pollution include polyamide, polyacrylonitrile, polyester, polyvinyl chloride [72] and sulphur-based polymers [73,74]. Multiple studies have been conducted for introducing polyamide-based highly efficient, thermo-mechanically stable pervaporation membranes [75,76,77,78,79,80,81,82,83]. The production of polyamide is around 8.5 million/tons per year [81] and expected to reach 10.4 million tons by 2027 [84,85]. Polyamide is synthesized by two different routes, i.e., ring opening polymerization [86,87,88] and step-growth polycondensation [89,90]. Starting substrates involved in both of these processes are obtained by the petrochemical industry [91]. Polyamides contain heteroatoms (nitrogen and oxygen of amide linkage –CO–NH– ) in their main chain, which does not solely consist of C–C bonds. They are prone to hydrolytic degradation under certain conditions due to the hydrophilicity of the amide linkage [92]. However, their stay in landfills ranges from decades to centuries [93] because of some reasons, i.e., crystallization, hydrophobicity and limited accessibility of enzymes, which hinder biodegradation [94]. Some studies reveal that limited effective enzymes have been discovered for the biodegradation of high molecular weight polyamides so far [95,96,97,98]. As microplastics, polyamide is a major environmental pollutant that accounts for 17% of sewage treatment plants in Italy [99] and almost 53% of Pikku Vesijävri pond and Vesijävri lake near Lahti city in Finland [100].

Polyamide microplastics are found in the bodies of aquatic organisms, e.g., almost 35.6% of ten species of fish from the English Channel [101], in the gastrointestinal tract of *Oreochromis niloticus* and Halibut nematodes [102,103], and in other animal species, e.g., almost 46.11% in the lungs of pigs [92]. Polyacrylonitrile used for the pervaporation desalination [104,105] is another plastic material having excellent weather resistance, and photostability because of which it persists for longer periods in the environment under normal conditions [106]. An example of studies investigating the detrimental effects of polyacrylonitrile on aquatic life is the work of Zhang et al. (2023). The results showed that polyacrylonitrile microfibers adhering to the zebrafish embryonic surface caused pericardial edema in larvae after hatching, increased the heart rate, changed the calcium ion pathways and fat metabolism, restricted the growth patterns and reduced the survival rates [107]. The presence of such microplastics has not only detrimental effects on the health of such organisms but also causes variations in entire food chain patterns, which completely disrupts the natural equilibrium established in the ecosystem [108,109,110]. Some of the major health hazards to humans posed by such microplastics include organ damage, obesity, reproductive troubles and delay in child development [111]. Nowadays, the studies are oriented on the effects of microplastic pollution [111,112,113,114,115,116,117] and the introduction of suitable biodegradable alternatives for fossil fuel-based hazardous polymeric materials [118,119] in order to minimize the eco-toxicity [120,121,122,123,124]. A considerable reduction in carbon footprint is offered by such materials as the green origin, i.e., plants capture atmospheric CO_2_ compared to fossil fuels, which tend to release CO_2_ in the ecosystem [125,126,127]. Another decisive factor in this regard is the limited availability and the corresponding increase in prices of fossil fuel-based materials [86,128], which draws the attention of researchers towards the renewable green origins.

## 4. Pervaporation Membranes Made from Biodegradable Polymers

Considering the challenges of using fossil fuel-based polymers for pervaporation desalination, i.e., pollution, health hazards, dependence on fossil resources and price volatility, the trends are shifting towards biodegradable materials. In the marketing of biopolymers, Europe, North America and the Asian-Pacific areas are at the top [129]. Since the very beginning of civilization, the extensive use of biobased materials by mankind cannot be denied. Different biologically originated materials have been used for various applications such as leather, wool, fur and leaves for clothing, amber and bones for jewellery, wood for construction and tools. However, with the advancement of living standards, applications are also modernized, for instance, the use of biobased materials for membrane (plastics) fabrication for various separation purposes. Many widely studied membrane-forming biopolymers are polysaccharide-based. Biodegradability of biopolymers is one of the key factors behind their hype, as it is synchronized with the goal of pollution reduction caused by membrane materials. As green membranes, biopolymers are considered sustainable materials to be used for different purification and separation goals [130].

### 4.1. Polyvinyl alcohol Membranes

A widely studied hydrophilic polymeric material for pervaporation desalination is polyvinyl alcohol, which is synthesized by hydrolysis of polyvinyl acetate and is a semi-crystalline white powder [131]. The intrinsic properties of polyvinyl alcohol that make it an attractive and promising candidate for pervaporation desalination include high hydrophilicity that enhances the selective permeability of water molecules, eco-friendliness based on its biodegradability, tunable structure for functionalization [132], non-toxicity, moderate chemical stability and good film-forming properties [133,134]. Biodegradation of polyvinyl alcohol was discovered for the first time in 1936, where a phytopathogenic fungal species, *Fusarium lini,* yielded carbon dioxide and water as a result of degradation in the presence of dehydratase enzyme [135]. Other organisms that enzymatically degrade polyvinyl alcohol via various routes and use it as a source of carbon include *Pseudomonas* [136,137,138], *Alcaligenes, Bacillus* [139], *Saccharomyces*, *Rhodotorula*, *Endomyces*, *Pichia*, *Zigosaccharomyces*, *Nadosonia* species [139,140] and *Brevibacterium incertum* [141]. Some studies revealed that certain microorganisms in symbiotic relationships also tend to utilize polyvinyl alcohol to meet their nutritional demands [142,143,144,145]. It has been reported that only 46 days are required for the degradation of 75% polyvinyl alcohol in the presence of microorganisms containing suitable enzymes [139], which ensures a quick and benign biodegradation of polyvinyl alcohol compared to ecotoxic plastics. However, biodegradable synthetic polymers, including polyvinyl alcohol, are comparatively more expensive than petroleum-based alternatives such as polypropylene and polyethylene. The current economic assessments exhibit a good cost-to-performance balance of polyvinyl alcohol materials in terms of their mechanical strength and durability compared to fossil fuel-based alternatives, which are two to three times cheaper [146]. Elgharbawy et al. (2024) suggested the blending of polyvinyl alcohol with natural biopolymers having hydroxyl groups and required structural compatibility, i.e., starch, chitosan and carboxymethyl cellulose [147] in order to reduce the overall cost as well as to enhance the extent and rate of biodegradability [148]. Therefore, polyvinyl alcohol blended with an inexpensive natural biopolymer appears as a promising sustainable material for pervaporation desalination membranes.

### 4.2. Cellulose Membranes

Cellulose and its derivatives were among the first materials used for membrane fabrication because of their attractive features, including availability, biocompatibility, biodegradability, water permeability, hydrophilicity and robustness [149]. At first, cellophane, a cellulosic product, was employed for the production of hemodialysis membranes. Plants are the main source of cellulose. The linear arrangement of monomeric saccharide units establishes a fibrous configuration of polymer. The structural and chemical variants of cellulose are prepared with desirable features for specified applications by modifications in the parent polymeric chains having hydroxyl moieties [130]. Cellulose derivatives employed for effective pervaporation desalination include cellulose acetate, cellulose diacetate, cellulose triacetate, carboxymethyl cellulose and other forms of native cellulose (e.g., cellulose nanofibrils, cellulose nanocrystals, bacterial cellulose) [149,150,151,152,153]. Among all of these cellulose derivatives, cellulose triacetate is widely used in desalination membranes [11,154,155,156] and other industrial applications such as gas permeation [157,158,159], oil/water separations [160,161,162], hemodialysis [163], adsorptive removal of heavy metals [164,165] and removal of viruses [166]. In the context of biodegradability, cellulose, as a natural linear polysaccharide, is readily susceptible to enzymatic hydrolysis by cellulase in various environments. However, the degradation of acetate derivatives of cellulose is complex and depends upon certain environmental (temperature, pH, presence of microorganisms, moisture, availability of oxygen, nitrogen starvation, location and sunlight) and structural factors (molecular weight, size, extent of crystallinity, degree of substitution, physical form, redox potential and presence of contaminants). In the case of cellulose acetate, the rate of degradation decelerates with the increase in degree of acetylation [167]. The presence of deacetylating enzymes along with cellulase is required for the degradation of acetylated derivatives of cellulose [168,169,170]. In a more general perspective, a trade-off exists between the thermo-mechanical stability and biodegradability of cellulose-based membranes. Despite various advantages offered by cellulose-based membranes, the application of such materials for desalination faces some sustainability challenges in their preparation and application, i.e., non-ecofriendly and energy-expensive preparation of raw materials, lack of efficient green solvents and short lifespans [171]. The industrial-scale synthesis of cellulose via oxidation and acid/alkali treatment of plants [172] is a pollution-causing and expensive procedure [171]. The industrial scale processing of cellulose derivatives, i.e., cellulose triacetate, follows a heterogeneous derivatization, which is a complex, energy-intensive, chemically demanding and time-consuming reaction [173,174,175]. The cost analysis exhibits that the production of cellulose esters from plants or petrochemicals costs 4.0–20.0 €/kg, which is more expensive than petroleum-based alternatives [176]. Another challenge is the poor solubility of cellulose because of its compact hydrogen bonding network and high crystallinity. The solvents that show good to fair solubility of cellulose are toxic and cause pollution. The complex process of recovery and expensive materials are hurdles for large-scale applications of ionic liquids, emerging solvents with a high solubility potential for cellulose [177,178]. In the case of cellulose derivatives, the trend of green solvents cannot be applied on a wider scale because of limited solubility. Even the solvents that offer higher solubilities show processibility limitations, which hinder the preparation of casting solutions and, in turn, membrane fabrication [171]. Recent findings by Kim et al. (2024) demonstrate that the casting solutions prepared using green solvents, i.e., rhodiasolv polarclean and cyrene with 12 wt% cellulose acetate, were too viscous to yield uniform membranes on the laboratory scale [179]. All of these challenges need to be addressed to advance with cellulose-based membranes in a more sustainable manner.

### 4.3. Chitosan Membranes

Since the 1980s, chitosan has been employed for various separation applications [180]. Chitosan is a linear copolymer of β-(1,4)-linked D-glucosamine and N-acetyl-D-glucosamine units [181,182,183]. After cellulose, the second most abundant polymer in nature is chitosan, which can be obtained by the deacetylation reaction of chitin. Chitin is abundant in nature in the cell walls of some bacterial and fungal species and predominantly in the exoskeleton of insects and crustaceans. Chitin is a linear polysaccharide composed of β-(1,4)-linked N-acetyl-D-glucosamine [184]. Chitin and chitosan-based membranes have been employed for various applications, including oil/water separations [185], wastewater treatment [186,187], removal of dyes [188] and ion exchange separations [189,190,191]. Due to the high hydrophilicity of chitosan, it is considered a suitable candidate for pervaporation desalination applications and shows an excellent performance [56]. The extent of biodegradability of chitosan enhances with the increase in its degree of deacetylation, which ranges from 70 to 90% based on its application in various industries [192,193]. The microbial degradation of chitosan yields simpler products that are even more susceptible to degradation than the parent molecule [194]. The enzymatic affinity of chitosan decreases with the increase in molecular weight and vice versa [195]. The enzymes that are involved in the degradation of chitosan include lyases and hydrolases [196]. In marine ecosystems, degradation of chitosan via chitosanase enzyme yields oligomeric glucosamine, which acts as a direct source of nutrition for microbes [196,197]. In a terrestrial environment, the complete degradation of chitosan/polyvinyl alcohol films via *E. coil*, *S. aureus* and *Aspergillus niger* occurs in 21–28 days [198,199]. The degradation products of chitosan, i.e., N-acetyl glucosamine and D-glucosamine, have limited toxicology records. The European Chemical Agency has declared N-acetyl glucosamine to be a non-hazardous material [196]. Despite their various advantages (biodegradation, low toxicity levels, high hydrophilicity, etc.), the purification and processing expenses of biopolymers leave room for further research and innovation.

### 4.4. Poly(lactic acid) Membranes

Poly(lactic acid) is an aliphatic thermoplastic biodegradable polyester with a simple chemical representation as (C_3_H_4_O_2_)_n_ [200], which is based on lactic acid or 2-hydroxypropionic acid (C_3_H_6_O_3_) monomer.

The polymerization of lactic acid monomers yields poly(lactic acid), as given by Carothers equation [201]:

(1) $$\bar{X_{n}\;}=\frac{1}{1-p}$$

where p represents conversion upon polymerization and $\bar{X_{n}\;}$ is the average value of the degree of polymerization.

Poly(lactic acid) has numerous advantageous features that make it a remarkable material for membrane applications [66,67,202,203]. Nowadays, the widespread applications of poly(lactic acid) mainly rely on its relatively fast biodegradation under composting conditions, as compared to complicated degradation procedures of traditional petroleum-based alternatives [204,205]. Its degradation procedure yields CO_2_ and H_2_O, which are employed in pharmaceutical applications focused on drug delivery or scaffolding [206]. As a non-polluting plastic, its films and coatings are used in various industries, including food, packaging, agriculture, electronics, etc. [207,208].

#### 4.4.1. Synthesis of Poly(lactic acid) Through the Lens of Green Chemistry

**Figure 2:**
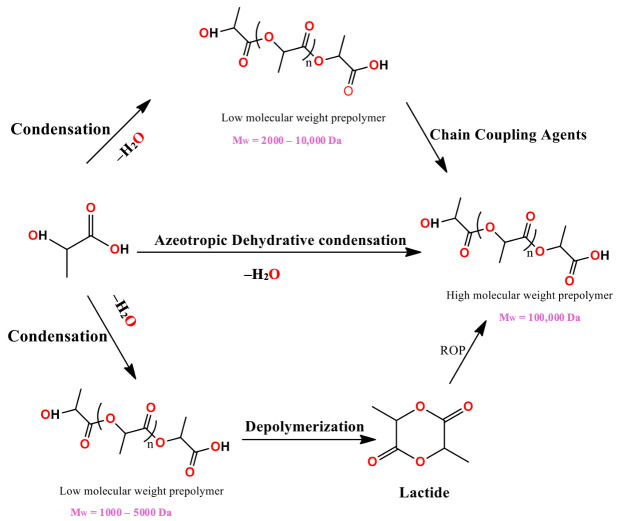
Synthesis Scheme of High Molecular Weight Poly(lactic acid) [209].

The synthesis of poly(lactic acid) itself adopts two alternative routes, i.e., melt polycondensation of lactic acid or ring opening chain polymerization reaction of lactide (Figure 2) [209].

However, poly(lactic acid) membranes are synthesized by various methods depending upon the application-based requirements. Phase inversion (non-solvent induced-phase inversion [210,211,212,213,214,215,216,217,218,219,220,221,222,223,224,225], thermally induced-phase inversion [226,227,228,229,230,231,232] and solvent evaporation induced phase inversion [233,234,235]) and different spinning modes (i.e., electrospinning [236,237,238], melt spinning [239,240], air jet spinning [241,242] and solution blow spinning [243,244,245]) are often used conventional synthesis modes, while 3-D printing [246,247,248,249] is a modern one that offers potential advantages such as structural control and design flexibility. Among all available techniques, phase inversion is commonly employed for pervaporation membrane synthesis (Table 1).

Despite its intrinsic biocompatibility, biodegradability and non-toxicity, poly(lactic acid) is not the only component to be considered, as the choice of solvent also plays a major role in membrane synthesis and cannot be neglected. The list of currently used toxic conventional organic solvents for poly(lactic acid) includes 1,4-dioxane, chloroform, dimethyl formamide, dimethyl acetamide, etc. Nowadays, the production and management of large volumes of contaminated solvent waste streams generated upon the usage of such solvents is a global challenge [250,251,252]. Moreover, these solvents are considered extremely hazardous not only for the environment but also for human health. Their commercial application has led to some incidents in the recent past. For instance, in Zhuhai city (Guangdong province, China), in August 2023, an incident of dimethyl acetamide poisoning was reported at a spandex production unit. The workers dealing with the polymerizers cleaning task were hospitalized for severe nausea and vomiting complaints due to a prolonged dimethyl acetamide exposure [253]. The European Chemicals Agency has already included such solvents in the list of chemicals that are intended to be banned [254]. It is also evident that the application of these solvents is not synchronized with the ‘Twelve Principles of Green Chemistry’ by Anastas and Warner (1998) [235]. Therefore, it draws the attention of researchers towards eco-friendly, potential green alternatives to dissolve poly(lactic acid), such as ethyl lactate, methyl lactate, cyrene, dimethyl isosorbide, gamma valerolactone [61] (Table 2) and acetyl tributyl citrate [231]. A recent study by Boura et al. (2024) provides a good basis to dig into the Hansen solubility sphere of poly(lactic acid) and the selection of desired solvents as per requirements [255].

#### 4.4.2. A Critical Balance: Application-Required Stability and Eco-Friendly Degradability of Poly(lactic acid) Membranes

All biodegradable polymers and their membranes degrade: (1) under specific conditions (humidity, temperature, light of a specific wavelength, aerobic/anaerobic environment, presence of certain species of microbes,,, etc.) [256,257,258,259,260], and (2) based on their physicochemical nature (molecular weight [246], extent of crystallinity [261,262,263], structure and morphology [264], presence of additives and copolymers [265,266,267], nanofillers [268], hydrophobicity [269] etc.). Hence, poly(lactic acid) undergoes degradation in many ways: (a) hydrolytic degradation, (b) thermal degradation, (c) photodegradation and (d) biodegradation [200]. All these mechanisms are interlinked and make it a complicated process overall. An easy degradation of poly(lactic acid) and its membranes (under required conditions) to simple, non-toxic mineralization products (CO_2_ and H_2_O) is environmentally favourable or environmentally benign. However, at the same time, it is a major obstacle in terms of stability to extend their application in some domains, i.e., those requiring a high operational temperature. Multiple studies have been conducted in order to enhance the stability of poly(lactic acid) membranes to an optimal extent by varying key parameters, and their effect on the rate of degradation was reported (Table 3). Such studies serve to optimize various parameters for a specific application while considering the eco-friendly aspects and degradation.

(a) Hydrolytic Degradation Mechanism

In hydrolytic degradation, polar ester bonds (carbonyl groups) in the structure of poly(lactic acid) undergo random cleavage [200,279], depending upon (a) the amount of water absorbed, (b) kinetics or simply the rate constant of overall hydrolytic reaction, (c) solubility of final products, and (d) coefficient of diffusion of polymeric chain fragments [200]. Later on, the increasing number of carboxylic moieties with progressing hydrolysis auto-catalyzes a further degradation reaction at free chain ends [280]. In the case of a solid membrane matrix, two distinct hydrolytic degradation mechanisms are observed: (1) slower surface-dominated hydrolysis at temperatures below the glass transition temperature and (2) fast bulk hydrolysis at temperatures above the glass transition temperature.

(b) Thermal Degradation Mechanism

Thermogravimetric analysis of poly(lactic acid) revealed the initiation of thermal degradation at temperatures higher than 200 °C [281]. Three kinds of reactions are considered in thermal degradation: (1) trans-esterification reactions within the molecules, (2) radical reactions and (3) pyrolytic elimination reactions [282].

(c) Photodegradation Mechanism

Ultraviolet and gamma radiations have a chain scission effect on poly(lactic acid). Multiple ultraviolet degradation mechanisms have been reported [283]. Predominantly, photolytic or photo-oxidative degradation of poly(lactic acid) follows the Norrish II mechanism [200,276,277], where C–O bond cleavage leads to the formation of –OH groups and C=C bonds at the termination points. In other possible degradation pathways, different products are formed, such as hydroperoxide, –COOH groups, carbonyl radicals and anhydrides [200].

(d) Biodegradation Mechanism

The overall mechanism of poly(lactic acid) biodegradation involves two distinct stages, i.e., fragmentation and mineralization. (1) During fragmentation, hydrolytic depolymerization of poly(lactic acid) to lactic acid takes place [284,285,286]. It happens either biotically via microbes containing special degradation enzymes or abiotically via chemical weathering [286]. (2) During enzymatic mineralization, weathering takes place via microorganisms and yields simple degradation products, i.e., CO_2_, H_2_O and methane [286,287]. The nature of these end products is decided by the oxygen content of the environment. Based on the availability of oxygen, aerobic (compositing) and anaerobic (landfilling) biodegradation mechanisms are possible [200,288]. Therefore, two decisive factors in biodegradation are optimal environmental conditions (temperature, pH, etc.) [289] and the presence of suitable microorganisms [290]. While taking the environmental aspect under consideration, temperature plays a decisive role in determining the biodegradation rates of poly(lactic acid). Under typical environmental conditions (ambient temperature), poly(lactic acid) biodegrades extremely slowly or negligibly in case of both freshwater and seawater [258,291,292,293,294] due to two reasons: (a) The intrinsic hydrophobicity of material that reduces the chances of hydrolysis [295,296,297] and (b) The inactivity of poly(lactic acid) degradation enzymes in freshwater and seawater under typical conditions [294,298,299], which do not favour the production or enough production of required enzymes. Furthermore, the number of crystalline domains increases in poly(lactic acid) after hydrolysis, which offers resistance to its further degradation [200,287,288,289]. On the other hand, hydrolytic cleavage accelerates above its glass transition temperature (~55–60 °C) [300,301,302]. It happens because of the relatively fast diffusion of water molecules through amorphous regions [286,303]. The chances of microbial attachment also increase at high temperature [304]. However, the literature reveals that the degradation is limited in marine environments [286], which makes poly(lactic acid) a good material of choice for seawater desalination. The thermal resistance of the material could be increased by suitable modifications (for instance, the use of inorganic nanomaterials, e.g., alumino-silicate clay, halloysite [10]) for pervaporation. The enzymatic degradation of poly(lactic acid) is a slow process compared to the biodegradation of other polyesters [305]. 

Microorganisms that are responsible for the biodegradation of poly(lactic acid) contain either of the two kinds of depolymerase enzymes [306], i.e., protease [307,308,309] or lipase (cutinase) [310,311,312]. Proteases are efficient for the breakdown of poly(L-lactic acid), while cutinases are involved in the breakdown of poly(D-lactic acid) [306]. Multiple species of filamentous bacteria, i.e., actinomycetes [287,288], fungi [287,313], small insects, some annelids, e.g., *Lumbricus terrestris* (earthworm) [288] and some other bacterial strains [287,313] contain these enzymes (Table 4). They tend to degrade poly(lactic acid) in both liquid culture media and soil and compost [298]. However, the efficacy of microorganisms depends upon their activity level, which in turn depends on various other factors [287], e.g., pH [314], molecular weight of enzyme, choice of inhibitor [315], temperature [316], molecular weight of polymer [317] exposure duration of polymer with specific degradation enzyme [317,318], specific degradation mechanism followed by an enzyme [319,320], and superficial and physicochemical features of the given polymeric sample [320,321] etc. [317,321], 

## 5. Present and Future of Poly(lactic acid) Membranes for Pervaporation Desalination

### 5.1. Present: A Concise Comparison of Poly(lactic acid) with Other Pervaporation Desalination Materials

The use of poly(lactic acid) membranes for desalination applications, precisely via pervaporation, has been recently developed, so the literature is scarce. However, a brief comparison of operational parameters and the resulting performance of poly(lactic acid) membranes with other pervaporation desalination materials (i.e., polyamide, polyacrylonitrile, polysulfone, polyvinyl alcohol, cellulose and its derivatives, and chitosan) depicts an inverse proportionality in water flux values and increasing salt concentrations in the feed solution for all polymers, which is attributed to the reduced vapour pressure of water at higher salt concentrations (Table 5). Overall, the performance of poly(lactic acid) membranes is comparable to other polymers in terms of water flux and salt rejection at high feed salt concentration and reasonable operational temperature.

### 5.2. Future: Suggestions to Improve the Performance, Long-Term Stability and Commercial Applicability of Poly(lactic acid) Membranes

In commercial settings, biobased poly(lactic acid) is obtained from natural sources, e.g., Natureworks’ Ingeo polymer, which is derived from fermented corn starch sugars and consists predominantly of poly(D-lactic acid) and poly(L-lactic acid). Higher ratios of poly(L-lactic acid) are beneficial because of its attractive features, i.e., higher melting point (175 °C) and glass transition temperature (60–65 °C), good crystallinity (37%), high modulus (~3.8 GPa), and good tensile strength (~59 MPa) [332,333,334]. Moreover, the degradation of poly(L-lactic acid) is slower than some other biodegradable polymers, i.e., poly(ε-caprolactone), which contributes to its sufficient stability for desired application durations. This feature is attributed to the delay in initiation of hydrolytic cleavage caused by the bulk of methyl entities in the backbone, imparting hydrophobic character. The stability of high molecular weight poly(lactic acid) is evident from the persistence of its implants up to 2–5.6 years until their complete *in vivo* degradation [333,334]. Generally, the degradation rate is highly dependent on several factors, i.e., extent of crystallinity, thickness, porosity and surrounding environment. However, there are limited studies focused on long-term stability relevant to the commercial scale applicability of poly(lactic acid) membranes for desalination, leaving room for further exploration. A few available findings to enhance the long-term stability of poly(lactic acid) membranes aid in the optimization of various structural and operational parameters in the given aqueous environment for pervaporation desalination application. Such studies suggest some modifications (additives, nanofillers, choice of coagulation bath and blending of poly(lactic acid) with other polymers, etc.) that could play a significant role in the improvement of performance parameters as well as stability of poly(lactic acid).

#### 5.2.1. Extent of Crystallinity

The studies exhibit a significant decrease in the overall rate of biodegradation and a corresponding increase in the mechanical strength with the increasing number of crystalline domains in a polymeric framework. This happens because of the fast hydrolytic degradation of amorphous chains compared to crystallites [335]. As the growth of crystallites involves the alignment and organization of poly(lactic acid) chains along the crystal lattice, the more orderly growing crystallite in the polymeric framework restricts the mobility of amorphous chains. Simply, restriction in mobility is caused by the occupation of previously available free volume by highly packed crystalline chains. Moreover, the higher melting point of highly crystalline polymeric frameworks also imparts deformation resistance at elevated temperatures [336].

##### Role of Additives

In general, crystallization of poly(lactic acid) takes place via heterogeneous nucleation, while homogeneous nucleation is rare because of the absence of reacting side chain groups [335]. In order to achieve an optimal degree of crystallization, the molecular movement of amorphous chains requires high energy. This minimum energy barrier to obtain an ordered crystal structure could be reduced by heterogeneous nucleation additives [337,338]. Such additives enhance both the performance and thermo-mechanical stability of the material [339,340], which plays a crucial role in high temperature applications, e.g., pervaporation desalination. For instance, in a study by Moriya et al. (2009), the effect of polyethylene glycol addition on the filtration performance of poly(lactic acid) based membranes was investigated. The results exhibited a significant increase in water permeability with the increasing ratios of the additive. The highest water flux of 882 kg/m^2^h was obtained with 10 wt% polyethylene glycol [341]. However, the addition of polyethylene glycol up to 3–5 wt% is usually advised for the fabrication of poly(lactic acid)-based dense membranes [10]. An increase in the concentration of polyethylene glycol increases the extent of crystallinity and reduces the viscosity of the solution up to 10 wt% addition [341]. In another study, Battegazzore et al. (2011) examined the crystallization kinetics and their effect on the stability of poly(lactic acid) upon the addition of a mineral, talc (5–15 wt%). The results showed an increase in the degree of crystallinity of poly(lactic acid) up to 36.9% at the highest talc concentration (15 wt%) [342]. The studies employing such heterogeneous nucleation additives from various material categories, along with their mechanism of action and effectiveness to enhance the performance and stability of poly(lactic acid), are described below.

(a) Nanofillers

The addition of nanomaterials enhances the extent of crystallinity in poly(lactic acid). Because of their high surface area, nanoparticles act as effective nucleating agents with multiple nucleation sites. Such materials develop small but highly uniform crystallites [335]. In a recent study, Nigiz et al. (2023) employed modified poly(lactic acid) membranes having alumino-silicate clay, halloysite as a nanofiller for pervaporation desalination of seawater. The ionic exterior and interior containing hollow nanotubular structure of halloysite makes it a good choice for water treatment applications. At low prices, halloysite offers excellent physico-chemical features, mechanical stability, good miscibility and antimicrobial properties to the polymeric matrix. The effect of increasing halloysite concentration (0–5 wt%) was investigated at 40–60 °C operational temperature, 10–40 mbar pressure on the downstream side with 2–6 wt% of NaCl in the feedstream. Results showed a significant increase in the mechanical strength (from 4.46 MPa to 13.85 MPa), hydrophilicity, stability and desalination performance of poly(lactic acid) membranes with the increasing halloysite content. The 5 wt% halloysite-containing poly(lactic acid) membrane exhibited the highest water flux of 13.14 kg/m^2^h along with 99.5% salt rejection. Testing with seawater yielded water quality meeting the standards of drinking water. The highest seawater flux through modified poly(lactic acid) membranes was 10.4 kg/m^2^h with high salt rejection. Moreover, after testing for 100 h, the performance parameters were consistent. Simple water-washing was sufficient before reusing the same membrane sample. Excellent antibacterial properties were recorded in the antimicrobial testing [10]. The reason behind the hype of nanoclay in academia and industry is the presence of layered mineral silicates in its structure [343,344]. In another study by Day et al. (2006), the lamellar growth of poly(lactic acid) crystals along the surface of nuclei was reported upon the addition of nano-clay. The isothermal crystallization of poly(lactic acid) exhibited Avrami behaviour with an Avrami index (n) of two. The results obtained by the Avrami equation exhibited an immense increase (15–20 times) in the initial growth and nucleation density by the addition of nanoclay at 120–130 °C, while the crystallization half-time was decreased 2–3 times [345]. These studies demonstrate that the addition of nanofillers significantly improves the water permeability due to improved hydrophilicity and thermo-mechanical stability of poly(lactic acid) by accelerating the crystallization kinetics.

(b) Zeolites

The well-defined microporous structure of zeolite also provides initiation and growth sites for crystallization of poly(lactic acid). For instance, in a recent study by Wang et al. (2021), a significant increase in the tensile strength of poly(lactic acid) from 55.0 MPa to 77 MPa upon the addition of 8 wt% zeolite was reported [346].

(c) Organic Salts

Some organic salts, e.g., potassium and sodium salts of 5-dimethyl sulfoisothalate, basalt powder, zinc phenyl phosphate, are reported to be efficient for increasing the crystallinity of poly(lactic acid) [347,348]. Barczewski et al. (2020) employed a modified strategy by using both basalt powder (a mineral, 5–20 wt%) and potassium salt of 3,5-(bismethoxycarbonyl) benzenesulfonate (an organic salt, 1 wt%) to enhance the thermo-mechanical stability of poly(lactic acid). The results showed a notable increase in various parameters, i.e., crystallinity (55%), heat deflection temperature (>100%), hardness (35 MPa), tensile modulus (0.75) by the addition of 20 wt% basalt powder along with 1 wt% organic salt, while the highest impact strength (2 kJ/m^2^) by the addition of 5 wt% basalt powder with only 1 wt% organic salt [349].

(d) Hydrogen Bonding Materials

Materials having the tendency to form hydrogen bonds within their own structure or with the carbonyl groups of poly(lactic acid), i.e., amides, also enhance the crystallinity and, in turn, stability of poly(lactic acid). The interface provided by such bonding acts as a nucleation site for rearrangement and high packing of larger crystallites [335]. For instance, Tang et al. (2011) investigated the impact of ethylene bis(hydroxytertamide) on crystallinity and relevant stability of poly(lactic acid). The results showed a significant decrease in crystallization half-time from 18.8 minutes for neat poly(lactic acid) to 2.8 minutes in the presence of ethylene bis(hydroxystearamide) at 105 °C. Moreover, the degree of crystallinity and thermal resistance were improved significantly upon annealing for more than 5 minutes [350]. Some other amide agents, e.g., N,N′-ethylenebis(stearamide) [351], N,N′-bis(2-hydroxyethyl)terephthalamide [352], N,N′-ethylene(10-undecenamide) [353], N-aminopthalamide [354], multi-amide agents (e.g., fulvic acid amide [355] and humic acid amide [356]), N,N′,N″-tricyclohexyl-1,3,5-benzenetricarboxylamide [357], octamethylenedicarboxylic dibenzoylhydrazine [358] and tetramethylenedicarboxylic dibenzoylhydrazine [335] also play a similar role in enhancing the extent of crystallinity and stability of poly(lactic acid) [335].

Blends of Poly(lactic acid) with Other Polymers

The strategy to blend poly(lactic acid) with some other polymers, i.e., poly(butylene adipate-co-terephthalate), poly(butylene succinate), poly(ε-caprolactone) [265] and orotic acid aids to accelerate the crystallization kinetics of poly(lactic acid), where the added polymer acts as nucleation sites for the crystallization of poly(lactic acid). Hence, the thermo-mechanical stability of the material is enhanced by blending it with other polymers under appropriate conditions [335]. However, an appropriate balance of crystalline to amorphous regions within the polymeric structure is essential for making the material stable enough to achieve the desired applications for required durations.

Ideal Crystallization Temperature

A recent study by Ma et al. (2020) provides an explanation for the appropriate choice of crystallization temperature and its effect on the thermal stability of poly(lactic acid). The studies suggest that a crystallization temperature in the range of 110–130 °C assists in achieving a good thermal resistance. In this temperature range, the Vicat softening temperature of the material increases (from 51.6 °C to 64.9 °C) with a very slight decrease in its tensile strength. However, for a crystallization temperature above 130 °C, lower nucleation density results in coarser morphology, leading to a decrease in Vicat softening temperature (53.2 °C) and a further reduction in mechanical strength due to non-homogeneous distribution of amorphous and crystalline domains [339].

##### Annealing

Some post-modification processes, e.g., annealing (heat treatment), also play a significant role in the optimization of the degree of crystallinity and relevant stability of poly(lactic acid). In an annealing process, polymeric chains from a solid amorphous phase become relatively mobile at an ideal crystallization temperature, i.e., 110–130 °C, because of the availability of energy higher than the crystallization barrier. Subsequently, the enhanced molecular movements aid in the rearrangement and packing into crystalline domains, which increases the overall degree of crystallinity. During the gradual cooling stage, polymeric chains organize themselves further and yield a highly stabilized polymeric structure. Hence, the overall strength and stiffness of the material are enhanced [335,359]. For instance, Dias et al. (2012) [261] conducted a study to analyze the hydrolytic degradation of electrospun poly(lactic acid) mats with varying extents of crystallinity. Thermal treatment of poly(lactic acid) fibres aided in achieving different crystallinities ranging from 0 to 45%. An alkaline phosphate-buffered solution was employed as a medium for degradation reaction for a period of 20 weeks. Results exhibited a significant decrease in the average molecular weight of amorphous fibres, but only a minor change in overall sample mass. However, increasing crystallinity suppressed this effect. The degradation process was also responsible for the shifts in the morphological features of polymer mats. A decrease in porosity and an increase in fibre diamete resulted in a closely packed fibrous network, upon degradation [261]. In a recent study, Sayed et al. (2024) [360] introduced silica (SiO_2_) modified poly(lactic acid) membranes with enhanced hydrophobicity for desalination via membrane distillation. The interesting part of their work is the post-treatment of membranes, i.e., heat pressing followed by annealing, which improved the thermal stability and overall desalination performance of the membranes. Their work shows a significant approach towards more stable poly(lactic acid) desalination membranes [360]. However, hydrophobicity imparted by silica and heat-treatment can be neutralized by the addition of hydrophilic nanofillers for pervaporation desalination. Thermal treatment and heat pressing contribute to the thickening and fusion of fibres that reduce the porosity of the membranes and make them more stable. Contrarily, the agglomeration of nanoparticles tends to have an opposite effect [360]. In another study, Gao et al. (2024) [361] reported that the increase in annealing temperature from 80 °C to 110 °C increased the degree of crystallinity from 2.5% to 50.5% and decreased the crystallization half-time from 34.2 minutes to 2.14 minutes. Moreover, they investigated the combined effect of blending poly(lactic acid) with another polymer. The results exhibited that the addition of nucleating agents, i.e., orotic acid and N,N’-ethylenebis(stearamide), reduced the crystallization half-time by 0.6 minutes at 110 °C. The combination of ideal crystallization temperature along with specific nucleating agents is an efficient strategy to improve the crystallization kinetics and to reduce the annealing duration, annealing temperature and crystallization half-time [361]. However, multiple factors collectively play a role in the attempts to enhance the stability of poly(lactic acid) via annealing (initial state of polymer and the technique used to prepare the sample, annealing temperature and duration, etc.), which require optimization for a specific application.

#### 5.2.2. Choice of Coagulation Bath

During the synthesis of poly(lactic acid) membranes via nonsolvent-induced phase inversion, the coagulation bath plays a crucial role not only because of the sensitivity of the material but also in determining the final properties and stability of the obtained membranes. In a recent study, Naseeb et al. (2023) [362] prepared poly(lactic acid)-based dense membranes using nonsolvent-induced phase inversion for reverse osmosis desalination. The results showed a significant improvement in water flux and rejection with the increasing content of ethanol in the coagulation bath composition. The best performance was obtained with the coagulation bath composition of ethanol: water = 80 wt%:20 wt% [362]. Despite the achievement of excellent performance using dense green membranes, the only concern that remains is the increased volumes of ethanol-containing wastewater. Xing et al. (2013) [219] also employed the ethanol/water coagulation bath for the fabrication of poly(lactic acid) membranes in order to investigate the effect of the choice of non-solvent on the porous framework, structure and crystallization of the membrane. They varied the concentration of water and ethanol in order to evaluate the best coagulation bath composition to get the required membrane morphology. The results demonstrate that a high concentration of water (low concentration of ethanol) leads to non-homogeneity in finger-like projections, reduced crystallinity and porosity and vice versa [219]. However, controlling the composition of the coagulation bath with high precision is challenging. In case of more accuracy, this method could be applied to obtain membranes with high mechanical strength due to the enhanced crystallinity.

#### 5.2.3. pH

In aqueous conditions, the plasticizing effect of water causes the biopolymers to lose some of their mechanical strength in the initial six months, and this trend declines afterwards. In case of hydrolytic degradation of poly(lactic acid), pH plays a crucial role [334,363]. A recent study by Vaid et al. (2021) [363] depicts a fast degradation of poly(lactic acid) in a highly alkaline environment (at pH 10). A significant mechanical strength loss (40%) was reported despite the high crystallinity of the samples. However, the change was minimal in the range of 2–7.4 pH [363]. Therefore, poly(lactic acid) could be a material of choice for seawater desalination (7.5–8.4 pH) with some additives and optimal operational parameters that enhance the hydrolytic stability of the material. The generally used hydrolytic inhibitors for such polymers are the compounds that are capable of interacting with both superficial carboxylic groups and moisture, e.g., carbodiimides [364]. However, carbodiimides are required in considerable concentrations (>1.25%) to enhance the stability of poly(lactic acid) and are comparatively expensive additives [365,366]. In a recent study, Hallstein et al. (2024) [366] introduced an aziridine derivative along with an acid scavenger as an alternative to carbodiimides. The acid scavenger alters the pH of incoming moisture and delays its interaction with the polymeric chain as well as the stabilizer, which helps in longer additive retention. The use of a small amount of stabilizer (0.5 wt%) along with an acid scavenger (1.5 wt%) delayed the hydrolytic degradation from 2 days for neat poly(lactic acid) to 49 days in the presence of additives at 60 °C. The problems associated with the negative impact of the hydrolytic inhibitor on crystallization and the high cost could be mitigated by the use of a small amount (0.5 wt%) [366]. Another study was also conducted by Hallstein et al. (2024) [367] to evaluate the thermal stability imparted by this inhibitor. The results depicted a significant increase in thermal ageing stability at 100 °C (from 500 to 600 h for neat poly(lactic acid) to 2000 h for the sample having an inhibitor) and at 150 °C (250 h for the sample having an inhibitor) [367]. Therefore, the addition of minute amounts of inhibitors could significantly improve the hydrolytic and thermal stability of poly(lactic acid) for high-temperature long-term applications in aqueous streams.

#### 5.2.4. Thickness and Porosity

The structural and superficial properties of dense poly(lactic acid) membranes prepared by phase inversion are predominantly dependent on multiple factors, e.g., evaporation time. A study by Galiano et al. (2019) [368] provides an explanation for the effect of such parameters on the final properties and performance of poly(lactic acid) pervaporation membranes prepared using a green solvent, i.e., ethyl lactate. The results exhibited that longer evaporation time (up to 7 minutes) resulted in denser membranes with reduced thickness and porosity but improved mechanical strength. Moreover, the membrane was highly selective (α of 75) for the separation of an azeotropic mixture (methanol/methyl-ter-butyl ether) [368]. This study significantly contributes to the idea of employing green solvents for the synthesis of dense poly(lactic acid) membranes via phase inversion with longer evaporation duration to obtain the membranes with low porosity, high mechanical strength, appreciable selectivity and a high flux for polar molecules.

#### 5.2.5. Fouling Resistance

Fouling is another challenge while dealing with polymeric membranes that can be mitigated by some simple modifications to increase the shelf-life of membranes. There exists a high probability of interaction of water molecules with polar oxygen atoms of ester linkages in the backbone of poly(lactic acid) and also with the additional –COOH and –OH on the terminals. This imparts a natural hydrophilicity to poly(lactic acid), which in turn reduces the chances of fouling [369,370]. Furthermore, some additives, e.g., sepiolite functionalized by silver (Ag) and copper (Cu), tend to enhance the antifouling potential of poly(lactic acid) membranes against bacterial (*Pseudomonas putida*) and yeast (*Saccharomyces cerevisiae*) species [371]. The loosely packed extensive capillary framework and the presence of hydrophilicity imparting surface silanol groups (–SiOH) in sepiolite make it an ideal choice to cope with biofouling. A strong hydrogen bonding interaction between the hydroxyl groups of sepiolite and the carbonyl group of poly(lactic acid) contributes to the uniform dispersion of sepiolite in the polymeric matrix. The negative charge of sepiolite hinders the attachment of microorganisms. Almost double permeability was reported in the case of metal (Ag or Cu) functionalized sepiolite compared to neat poly(lactic acid) membranes as a function of pH and, in turn, the availability of ions [341,371]. Some other biobased pore-forming agents, e.g., β-cyclodextrin along with a crosslinking additive, also impart antifouling potential to poly(lactic acid) membranes. For instance, Xiong et al. (2016) [372] synthesized poly(lactic acid) membranes via nonsolvent-induced phase inversion to investigate the effect of surface crosslinking and addition of β-cyclodextrin on antifouling potential, thermal stability and permeability of membranes. The poly(lactic acid) membrane surface was crosslinked with a copolymer poly(vinylpyrrolidone-vinyltriethoxysilane). The results showed an improvement in thermal stability and permeability for modified membranes [372]. In another study, Shen et al. (2013) [373] synthesized poly(lactic acid)/poly(lactic acid)-polyethylene glycol-poly(lactic acid) hollow fibre membranes to investigate the effect of chain length of additive (polyethylene glycol) on the fouling behaviour of membranes. Their results exhibited an increase in antifouling potential and a decrease in water contact angle with increasing chain length of polyethylene glycol because of high additive retention and increased residual ratio (up to 40%) [373]. Their work contributes to the idea of enhancing the permeability and antifouling potential of poly(lactic acid) membranes via blending with other polymers, which would be highly beneficial in applications like pervaporation desalination.

In general, the addition of various additives imparts hydrophilicity, mechanical strength, antifouling potential, high water permeability and good salt rejection to pervaporation desalination membranes. Blending of poly(lactic acid) with other synthetic and biosourced polymers is also a significant method to deal with all sorts of challenges. All of these strategies are the need of the hour, but the preference should be given to the eco-friendly additives and biosourced polymers to produce blends. The argument about the use of non-biodegradable additives and copolymers remains unresolved, as not many alternatives with better results have been introduced yet. On the other hand, the addition of non-ecofriendly materials to green membranes is a cause of secondary pollution. A biosourced polymeric membrane matrix degrades, leaving behind the undegraded additives in the environment that remain there for longer durations [374,375]. Despite their very small amounts, such non-biodegradable toxic additives are increasing day by day in the environment with the rapidly increasing production, testing and large-scale implementation of membranes globally. Another concern is the fair water solubility of many biopolymers. Additives in degraded biopolymers contribute to the aquatic pollution both in surface and ground resources and have an adverse effect on aquatic life as well as the quality of water [375].

## 6. Ecotoxicology of Poly(lactic acid)

### Poly(lactic acid) Microplastics: Role and Remedies

Some authors suggest that neat poly(lactic acid) may generate microplastic fragments during intermediate stages of degradation, so it cannot be a completely degraded polymeric material in the natural environment and may take several years to degrade [292,376,377]. For instance, limited enzymatic activity towards poly(lactic acid) in marine environments restricts the complete degradation of poly(lactic acid). It means that mainly amorphous domains are degraded, and crystalline ones persist longer in the environment, leading to the formation of microplastics [286]. Some recent studies also revealed that poly(lactic acid) exhibits significantly lower microplastic generation compared to polypropylene by a factor of 18 [378]. However, there are other studies that show the opposite results depending upon the experimental conditions [379,380]. The poly(lactic acid) microplastics are considered to have health effects on some aquatic species (e.g., fish, mollusks, annelids, zooplanktons, phytoplankton, algae and microorganisms). Additionally, they tend to reach the highest trophic levels of the food chain (humans) by long-term stay in the environment [286]. In contrast, some studies also revealed that the presence of poly(lactic acid) in some aquatic species is completely benign to their bodily mechanisms and their ecosystem functioning patterns [324,381]. In case of some microorganismic species, poly(lactic acid) microplastics were found to promote two main stages of the nitrogen cycle, which shows that these microplastics could even be wisely used as an energy source for such microorganisms [382] (Table 6).

Application of materials that can enhance the biodegradation rate of poly(lactic acid) would be an appreciable solution, not only considering the negative impact of poly(lactic acid) membranes on the environment and organisms, but also a good balance with performance parameters. Some of the desired materials to enhance the biodegradability and, in turn, reduce the long-term stay of its microplastics in the environment are:

(a) Hydrophilic polymers to prepare blends: Considering the application for pervaporation desalination, blending of poly(lactic acid) with other hydrophilic biopolymers would not only enhance its hydrophilicity or water diffusion for biodegradability but also participate in the performance. For instance, Wilfred et al. (2018) used starch as the polymer with poly(lactic acid), and the material showed biodegradation in just 14 and 28 days in compost and soil, respectively [391].

(b) Hydrophobic polymers to prepare blends: Blending of poly(lactic acid) with hydrophobic polymers could result in both ways, depending upon the chosen polymer, i.e., increasing the stability (e.g., poly(ε-caprolactone) [265]) or increasing the chain mobility (e.g., poly(vinyl acetate) [392]). In case of enhanced hydrophobicity, the water permeation through the membrane would be decreased, even though the membranes would be more stable.

(c) Additives: (1) Plasticizers: Many plasticizers are reported to contribute to accelerating the biodegradability of poly(lactic acid) by reducing the elasticity modulus and glass transition temperature of the material, e.g., polyglycerol [393] and acetyl-tri-*n*-butyl citrate [394]. (2) Others: Some other additives, i.e., biocatalysts, are also employed for this purpose [285].

## 7. Cost Comparison of Poly(lactic acid) with Other Biobased and Petroleum-Based Polymers

Poly(lactic acid) (2.8–3.5 €/kg) is the most cost-effective polymer compared to some other available biodegradable materials such as cellulose ethers (4.0–20.0 €/kg) [395] and polyhydroxyalkanoates (3.7–13.9 €/kg). On the other hand, like all biopolymers, it is an expensive choice compared to polluting and toxic petroleum-based alternatives such as polyethylene (1.3–1.7 €/kg), polyethylene terephthalate (1.19–1.2 €/kg) and polypropylene (1.7–2.0 €/kg) [395]. Overall, it is evident that poly(lactic acid) is a promising sustainable material with increasing economic competitiveness for large-scale applications. However, the raw material processing, modification (chemical and physical: to enhance the thermo-chemical stability and performance) and reduction in operational costs could be achieved by further research and optimization.

## 8. Conclusions

Desalination technologies offering freshwater to 4% of the global population include both thermal and membrane-based modes, where the latter are preferable due to simplicity, high performance, mild operational conditions, cost effectiveness and ease of scaling-up. Pervaporation desalination employing dense polymeric membranes is a promising technique for hypersaline desalination with excellent antifouling potential. Keeping in view the principles of green chemistry, biodegradable materials in combination with green solvents are an attractive choice for membrane fabrication. Multiple biodegradable materials have been reported in this regard, i.e., polyvinyl alcohol, cellulose and chitosan. The high cost (e.g., for polyvinyl alcohol and chitosan) and the low solubility in green solvents (e.g., for cellulose) are the two main problems encountered while employing green materials rather than cheaper petroleum-based toxic alternatives. Poly(lactic acid) is a promising polymer for pervaporation desalination based on hydrophilicity due to the presence of polar ester groups, high salt rejection when fabricated as dense membranes, biodegradability, sustainable production, low toxicity, low carbon footprint, easy processing and excellent solubility in green solvents. In comparison with other available biodegradable materials, poly(lactic acid) exhibits some advantages, i.e., (a) unlike cellulose, poly(lactic acid) is soluble in many green solvents, which helps in eco-friendly green synthesis of membranes (b) unlike chitosan, poly(lactic acid) offers a high chemical stability in hypersaline feed streams without a crucial demand of crosslinkers, greater dimensional and mechanical stability with minimal swelling in aqueous conditions and better processability and scalability, which are the keys to achieve a higher reproducibility in membrane fabrication and a stable long-term performance (c) poly(lactic acid) is more economical than polyhydroxyalkanoates. However, the challenges that are faced in its application are low hydrolytic stability at elevated temperatures, high cost, low biodegradability in natural environments and long-term persistence of its microplastics in the environment. The incorporation of minute quantities of additives such as plasticizers, nanofillers, crosslinkers and surface modifiers, blending of poly(lactic acid) with other polymers and post-treatments, e.g., heat pressing and annealing, result in a significant increase in the performance and offer a good balance in application-required stability and eco-friendly biodegradability of poly(lactic acid) films. It is also evident from the studies that poly(lactic acid) microplastics may cause health problems in some species but remain benign in others. The wise use of these microplastics as an energy source for microbes would help to accelerate the nitrogen cycle. Like all other biopolymers, the processing cost of poly(lactic acid) is required to be further optimized, even though the cost analysis shows it to be the most economical choice compared to others, i.e., polyhydroxyalkanoates. Therefore, the optimization of parameters contributing to the performance, thermo-mechanical stability for long-term operations, scalability using eco-friendly additives, and reduction in processing and operational expenses remains an important research direction in the near future.

## Figures and Tables

**Table 1:** Phase Inversion Methods for Pervaporation Membrane Synthesis.

Sr. No.	Method of Synthesis	Advantages	Disadvantages	Reference
1	Nonsolvent-induced phase inversion	- Room temperature processing - Versatility - Simplicity - Scalability	- An excessive use of strong and toxic solvents - Challenging management of large volumes of solvent waste after synthesis	[210,211,212,213,214,215,216,217,218,219,220,221,222,223,224,225]
2	Thermally-induced phase inversion	- Homogeneous porous framework of the membrane - Narrow pore size distribution	- Not suitable to produce membranes with pore size < 0.1 μm - Use of toxic solvents, e.g., phthalates - Challenging to maintain the temperature range	[226,227,228,229,230,231,232]
3	Solvent evaporation-induced phase inversion	- Yields membranes with desired uniformity in structure - Suitable for the synthesis of dense membranes - Easy and energy-efficient drying of liquid membranes at room temperature (for solvents with low boiling points) - Versatility - Simplicity	- An energy-intensive process if solvent evaporation via a vacuum oven for several hours is required (for solvents with high boiling points) - Long synthesis time for low-evaporative solvents	[233,234,235]

**Table 2:** Potential Green Solvents for Poly(lactic acid) Membrane Fabrication.

Sr. No.	Green Solvent	Chemical Formula	Boiling Point (°C)	Synthesis	Advantages
1	Ethyl lactate	C_5_H_10_O_3_	154	By the esterification reaction of lactic acid and ethanol	- An alternative to petroleum-based solvents without any compromise on functional efficacy - Completely eco-benign - Safe - Biodegradable - Low toxicity - Low viscosity - Low volatility - High poly(lactic acid) solubility comparable to acetone - Ability to form scaffolds and organogels - Wider liquid temperature range - Non-zone depleting - Recyclability
2	Methyl lactate	C_4_H_8_O_3_	144–145	By the catalytic esterification reaction of lactic acid and methanol	- Bio-based - Nontoxic - Eco-friendly - Half-life of photochemical degradation of methyl lactate vapours under aerobic conditions is only 144 h
3	Cyrene	C_6_H_8_O_3_	227	- By pyrolysis of cellulosic biomass - Best sustainable synthesis route employs hydrogen as a product of water splitting and no involvement of solvents	- Good alternative for NMP, DMAc and DMF due to similar features (water miscibility, polarity and density) - Ecologically safe degradation yielding CO_2_ and H_2_O - Resistance towards oxidative reactions - Stable at high boiling temperatures
4	Dimethyl isosorbide	C_5_H_10_N_2_O	222–240	- By methylation reaction of 1,4:3,6-dianhydro-D-glucitol (an anhydro sugar isosorbide) - One-pot cyclization methylation of D-sorbitol	- Water soluble - Non-hazardous - Inexpensive
5	Gamma valerolactone	C_5_H_8_O_2_	205–207	- Two-step synthesis reaction using lignocellulosic biomass - One-pot conversion of fructose to gamma valerolactone	- Low viscosity at ambient conditions - Low vapour pressure - Negligible inflammability - Excellent resistance towards decomposition reactions at high temperature in the absence of water - Stable for long durations in the presence of peroxides under ambient conditions, along with neutral or slightly acidic pH - Good alternative for acetone, ethyl acetate, *N*-methylpyrrolidone, *N*-ethylpyrrolidone and methylethylketone - Biodegradable (based on physicochemical features)

**Table 3:** Keys to Optimize the Application-Required Stability and Eco-friendly Degradation of Poly(lactic acid) Membranes.

Sr. No.	Goal of Study	Key Factor	Results	Reference
1	Analysis of hydrolytic degradation of electrospun poly(lactic acid) mats with varying extent of crystallinity	Crystallinity	Increased crystallinity decreased the degradation rate	[261]
2	Investigation of *in vitro* degradation of nanocrystalline electrospun poly(glycolide-co-lactide) membranes with varying mixing ratio of lactic acid and polyglycolide	Ratio of poly(lactic acid) in a polymer blend	Membranes with a low poly(lactic acid) ratio showed major weight loss after 144 h	[264]
3	Membrane degradation testing of porous membranes synthesized via freeze extraction method, using a blend of poly(L-lactic acid)/poly(ε-caprolactone) with variable mass contributions	Effect of co-polymer	- Poly(ε-caprolactone) addition to poly(L-lactic acid) pristine samples increased the stability - Poly(L-lactic acid) addition to poly(ε-caprolactone) pristine samples increased the degradation rate	[265]
4	Investigation of hydrolytic degradation of poly(lactic acid) membranes at varying pH	pH	- Alkaline pH favours degradation - Acidic pH also increases the degradation rate by favouring autocatalysis	[270]
				[200]
5	Investigation of thermal degradation of poly(lactic acid) membranes	Temperature	- Stable up to 200 °C for 20 min and only 1% weight loss afterwards - Either a two-step or single-step degradation in the range of 300–360 °C (unclear due to variation in stereochemical features and complexity of process)	[271]
				[272,273,274,275]
6	Investigation of photodegradation of poly(lactic acid) membranes by ultraviolet and gamma-rays	Radiations	- Degradation by UV-C radiations of 254 nm wavelength could be reduced by some stabilizers - *γ *-rays-based photodegradation yields ionic species	[200,276,277]
				[200,278]

**Table 4:** Microorganisms with Suitable Enzymes for Poly(lactic acid) Degradation [287].

Sr. No.	Target Poly(lactic acid) Isomer	Microorganisms Containing Suitable Enzymes	Examples	Reference
1	Poly(L-lactic acid)	(a) Actinomycetes	*Pseudonocardia alni* AS4. 1531(T)	[322]
			*Amycolatopsis* sp. strain HT32	[308]
			*Actinomedura* strain T16-1	[314]
			*Laceyella sacchari* LP175	[315]
			*Bacillus brevis*	[316]
			*Amycolatopsis orientalis* IFO12362	[317]
			*Saccharothix waywayaandensis*	[323]
			*Paenibacillus amylolyticus* strain TB13	[324]
		(b) Fungi	*Trichoderma viride*	[310]
			*Tritirachium album* ATCC 22563	[325]
			*Cryptococcus*sp. strain-S2	[312]
		(c) Other Bacterial Species	*Geobascillus thermocatinolatus*	[306]
2	Poly(D-lactic acid)	(a) Actinomycetes	*Bacillus stearothermophilus*	[307]
			*Paenibacillus amylolyticus* strain TB13	[324]
		(b) Fungi	*Aspergillus oryzae* RIB40	[311]

**Table 5:** Poly(lactic acid) Versus Other Pervaporation Desalination Materials in Terms of Operational Conditions and Performance Parameters.

Sr. No.	Membrane Material	Feed Concentration (wt% of NaCl)	Pervaporation Temperature (°C)	Water Flux (kg/m^2^h)	Salt Rejection (%)	Ref.
1	Poly(lactic acid) modified with 5 wt% Alumino-silicate clay, halloysite	4	60	11.9	99.95	[10]
2	Poly(lactic acid) modified with 5 wt% Alumino-silicate clay, halloysite	6	60	7.9	99.95	[10]
3	Polyvinyl alcohol/Polyacrylonitrile nanofibers	1.5	75	234.9	99.7	[40]
4	Surface-modified cellulose acetate	13.13	70	5.97	99.97	[49]
5	Chitosan/Graphene oxide mixed matrix membranes	5	81	30	99.99	[56]
6	Sulphonated styrene-ethylene/butylene-styrene copolymer	5	100	9.3	99.9	[73]
7	Sulphonated polystyrene-grafted poly(styrene-ethylene/butylene-styrene) block copolymer	5	75	76.8	99.95	[74]
8	Polyamide	20	60	8.4	99.9	[75]
9	Polyamide with polyacrylonitrile support	3.5	70	70	99.9	[76]
10	Polyamide with polyacrylonitrile support	10	70	40.8	99.9	[76]
11	Polyacrylonitrile/Kaolin mixed matrix membranes (PAN:Kaolin = 15:2)	3.5	65	82	99.93	[104]
12	Polyacrylonitrile/Kaolin mixed matrix membranes (PAN: Kaolin = 15:2)	10	65	59	99.91	[104]
13	Polyacrylonitrile (Hydrolysis post-treatment)	3.5	60	48	99.9	[105]
14	Polyvinyl alcohol/Polysulfone Composite	3.5	70	124.8	99.9	[134]
15	Polyvinyl alcohol/Polysulfone Composite	20	70	71.3	99.9	[134]
16	Polyvinyl alcohol/Polyacrylonitrile	3.5	70	32.26	99.98	[326]
17	Polyvinyl alcohol with polytetrafluoroethylene support	3.5	70	120	99.93	[327]
18	Polyvinyl alcohol with polytetrafluoroethylene support	20	70	51.95	99.93	[327]
19	Cellulose triacetate with 2 wt% Alumina loading	3	70	6.7	>99.8	[328]
20	Cellulose triacetate with 2 wt% Alumina loading	8.7	70	5.0	>99.8	[328]
21	Cellulose triacetate with 4 wt% LUDOX-Silica	3	70	6.1	99.8	[329]
22	Cellulose triacetate with 4 wt% LUDOX-Silica	5.9	70	4.8	99.8	[329]
23	Cellulose triacetate with 3 wt% cellulose nanocrystals	3	70	11.68	99.9	[330]
24	Cellulose triacetate (After alkaline post-treatment for 30 min)	8.7	70	107.5	99.9	[331]
25	Cellulose triacetate (After alkaline post-treatment for 30 min)	18.2	70	58.5	>99.8	[331]

**Table 6:** Impact of Poly(lactic acid) Microplastics on Different Species.

Sr. No.	Target Organism	Exact Species	Impact	Reference
1	Fish	- *Danio rerio* (Zebra fish)	- Microplastics accumulation in the epithelial lining of the intestine	[383]
			- Decreased intestinal pH	[384]
		- Zebra fish larvae	- Hindered skeletal development - Damaged mitochondrial morphology with relevant oxidative stress - Decreased swimming speeds	[385]
2	Mollusks	- *M. edulis* (Blue mussels)	- No impact on immunotoxicity	[324]
			- No impact on oxidative stress - Change in immunological response	[386]
		*- Mytilus edulis* (Blue mussels) and - *Ostrea edulis* (European flat oysters)	- No effect on eco-functioning	[381]
3	Annelids	- *Arenicola marina* (Lugworms)	- Reduction in feeding activity	[387]
4	Zooplankton	- *Artemia franciscana* (Crustacean)	- Accumulation in the digestive tract - Unaffected mobility	[388]
		- *Aurelia* species (Cnidarian—common jellyfish)	- Accumulation in gelatinous tissue - Unaffected mobility - Changed pulsation (swimming behaviour)	
5	Phytoplankton	(a complete natural community)	- Changed taxonomic composition - Number of cryptophytes raised as they have siliceous loricae, which protect against poly(lactic acid) microplastics - Cryptophytes were eliminated as they do not have any protection against poly(lactic acid) microplastics	[389]
6	Algae	- *Chlorella vulgaris* (Marine alga)	- Severe growth inhibitory rate of 47.95% - Increased cellular defence in stress situations by stimulating pigments (carotenoid, chlorophyll a and chlorophyll b)	[390]
7	Microorganisms	Sedimentary microorganismic communities	- Promoted nitrification and denitrification - Poly(lactic acid) microplastics are suggested to be used as a carbon or energy source	[382]

## Data Availability

Data are contained within the article.
